# Effect of home-based group conversation intervention using smartphone application on cognitive health and psychological well-being of older adults with subjective cognitive concerns in Japan: a randomized controlled trial protocol

**DOI:** 10.3389/fpsyg.2023.1114790

**Published:** 2023-05-16

**Authors:** Kumi Watanabe Miura, Seiki Tokunaga, Takuya Sekiguchi, Hikaru Sugimoto, Mihoko Otake-Matsuura

**Affiliations:** ^1^Cognitive Behavioral Assistive Technology Team, RIKEN Center for Advanced Intelligence Project, Tokyo, Japan; ^2^Japan Society for the Promotion of Science, Tokyo, Japan

**Keywords:** cognitive health, mobile applications, communication technology, social isolation, randomized controlled trial

## Abstract

**Background:**

Social activity is a key component in the prevention of cognitive decline. However, face-to-face social intervention has limited accessibility. To address this issue, we developed the “Photo-Integrated Conversation Moderated by Application” (PICMOA), a home-based group conversation intervention using smartphones. This paper introduces the PICMOA intervention and the protocol of the ongoing randomized controlled trial (RCT), which aims to evaluate the effects of PICMOA on the cognitive functioning and psychological well-being of Japanese community dwelling older adults at the risk of cognitive function decline.

**Methods:**

This study uses an RCT design in two parallel group trials with 1:1 allocation. The participants are community dwelling older adults aged 65 years and above, living in an urban city in Japan, with subjective cognitive concerns. In total, 81 participants were allocated to the intervention or control groups. The intervention group receives 30 min of weekly PICMOA sessions at their home for 12 weeks. The PICMOA intervention consists of (1) a photo preparation period before the session and (2) a structured group conversation session talking about the photos that participants took according to a specific theme. The control group receives 30 min of weekly health education videos on a tablet device. The primary outcome is cognitive functioning at pre- and post-phases of the 12-week intervention measured using the Telephone Interview for Cognitive Status in Japanese, semantic and phonemic fluency tests, and the Digit Span Forward and Backward tests. The secondary outcomes are psychological and social aspects including mental status, well-being, loneliness, and social support.

**Discussion:**

Interest is growing in internet-based activities for preventing social isolation. However, the effect of remote conversation interventions on cognitive functioning remains unclear. This study addresses this issue and provides a new avenue of social participation for older adults.

**Clinical trial registration:**

https://www.umin.ac.jp/ctr/, identifier: UMIN000047247.

## 1. Introduction

Cognitive health is a crucial concern for the health and well-being of older adults. Comprehensive interventions for modifiable risk factors could theoretically prevent or delay about 40% of dementias worldwide (Livingston et al., 2020). Social activity is one of the key factors for the prevention of dementia. There is vast empirical evidence on the longitudinal association between low levels of social activity and the occurrence of dementia (e.g., Fratiglioni et al., 2000; Karp et al., 2006; Saczynski et al., 2006; Amieva et al., 2010). A recent meta-analysis suggested that less frequent social contact, low social participation, and loneliness were significantly associated with the incidence of dementia with strengths comparable to other well-known risk factors of dementia (Kuiper et al., 2015). However, no global recommendation was made in the guidelines of the World Health Organization (WHO), as there was insufficient evidence that social activity intervention reduced cognitive decline and the onset of dementia to draw conclusions (World Health Organization, 2019). Therefore, it is hoped that the knowledge about the impact of social activities on cognitive decline will be accumulated through randomized controlled trials (RCTs).

One of the components of social activities is social contact in which people interact through conversations. There is a close association between linguistic ability, which is the underlying function of conversation, and cognitive health. Linguistic deficits often present in patients with mild cognitive impairment (MCI) and early stages of dementia (Taler and Phillips, 2008). Linguistic features may change as cognitive function declines. A previous study identified the characteristics of spontaneous speech among patients with mild to moderate Alzheimer's disease (AD), such as more production of word-finding delay, semantic paraphasia, empty and indefinite phases, and fewer repaired errors (Forbes-McKay et al., 2013). Another study from the Wisconsin Registry for Alzheimer's Prevention reported that features of speech fluency and semantic content in the spoken language declined faster among participants with early MCI than among those who were cognitively stable (Mueller et al., 2018). Some attempts have been made to screen patients with dementia and MCI by linguistic features such as speech volume, tense, and vocabulary (Fraser et al., 2016; Asgari et al., 2017).

In addition to linguistic ability, conversation is a task that stimulates multiple cognitive functions. Conversation requires attention, working memory, inhibition, and cognitive control (Ybarra et al., 2008; Ybarra and Winkielman, 2012) to read the intentions and feelings of others, maintain focus, manage issues flexibly, and control inappropriate behavior. Therefore, we hypothesized the benefits of conversation-based intervention on cognitive function. Based on our hypothesis, we developed our original intervention called Photo-Integrated Conversation Moderated by Robots (PICMOR), which is a structural group conversation program (Otake-Matsuura et al., 2021) and have examined the effect of this conversation-based intervention.

In the PICMOR program, participants have face-to-face conversation sessions in groups of four and talk about the photographs they took according to a pre-decided theme. As a result of utilizing PICMOR in an RCT among healthy older adults, phonemic fluency was significantly improved by following a 30-min weekly session for 12 weeks, compared to the control group in which free group conversations were held in the same frequency (Otake-Matsuura et al., 2021). Moreover, our post-intervention multimodal magnetic resonance imaging (MRI) showed significant differences in brain metrics between the PICMOR and control groups, which could be associated with the beneficial effect of the intervention on phonemic fluency (Sugimoto et al., 2020; Sugimoto and Otake-Matsuura, 2022a,b). For example, compared to the control group, the intervention group had higher resting-state functional connectivity between the left inferior frontal gyrus, one of the most important brain regions for verbal fluency, and right temporal pole which has been associated with semantic processing (Sugimoto et al., 2020). Additionally, resting-state functional connectivity between these regions was positively correlated with the enhanced phonemic fluency score measured by a neuropsychological test. Moreover, we attempted to elucidate neural underpinnings responsible for the intervention-induced enhancement of verbal fluency by analyzing structural characteristics, including brain volume (Sugimoto and Otake-Matsuura, 2022a) and diffusion tensor imaging (DTI) metrics (Sugimoto and Otake-Matsuura, 2022b).

Although the evidence of our intervention has been accumulated, such face-to-face interventions have one limitation: these are not applicable to the population at risk of isolation (e.g., disability, low accessibility area, or pandemic infection) and those who cannot physically access the intervention. In particular, the recent spread of COVID-19 has forced many older adults to become socially isolated. A prior report by Murayama et al. reported that the weighted prevalence (95% confidence interval) of isolation increased from 21.2% (20.7–21.7%) to 27.9% (27.3–28.4%) in Japan during COVID-19 (Murayama et al., 2021). Since social isolation is related to physical, mental, and cognitive health, there is a huge concern about the physical and mental health implications of social isolation and loneliness (Sepúlveda-Loyola et al., 2020).

In these situations, conversations are still possible even at remote locations using remote communication technology that facilitates widespread intervention. Therefore, we developed a smartphone application that provides home-based group conversation intervention through an easy interface, called “Photo-Integrated Conversation Moderated by Application” (PICMOA), based on our previous PICMOR intervention. In a similar study, Dodge et al. conducted an RCT that was a six-week intervention of talking to an interviewer via a PC-based video chat and found its short-term effects on the semantic and phonemic fluency compared to the controls (Dodge et al., 2015). Although there is a growing focus on internet-based social activity intervention to prevent social isolation, the effect of remote conversation intervention on cognitive functioning is still unclear.

Thus, we have conducted this trial to evaluate the effects of PICMOA on cognitive functioning and psychological well-being among older adults at risk of cognitive decline. Considering the aforementioned evidence, we hypothesized that remote conversations through the PICMOA intervention affects the maintenance of cognitive functioning. Additionally, we are interested in studying the effect of social interaction through the intervention on psychological well-being. In this paper, we introduce the overview of the PICMOA intervention and the whole picture of the ongoing RCT protocol utilizing the PICMOA intervention for 12 weeks. This trial uses an RCT design in two parallel group trials with 1:1 allocation and is registered with the University Hospital Medical Information Network (UMIN) clinical trials registry (UMIN000047247).

## 2. Methods

### 2.1. Study setting and eligibility criteria

**Figure 1:**
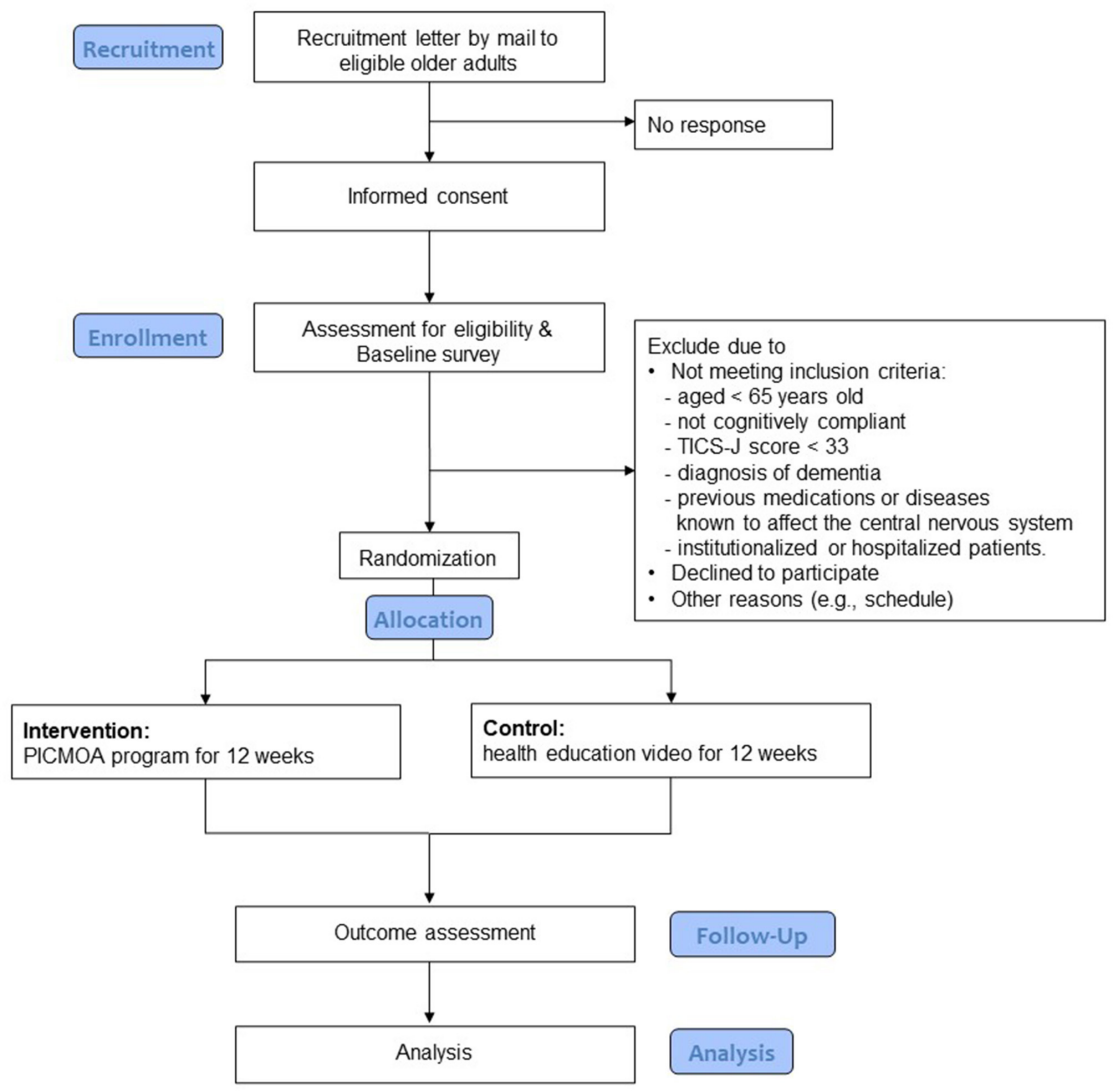
Process flow for participants in the study.

The participants are community dwelling older adults aged 65 years and above, living in Wako city, which is an urban area in Japan's Saitama Prefecture near the metropolitan area with a population of 83,821 as of March 2020 (Wako city, 2021). Figure 1 shows the flow of the trial for the participants, including the selection process. In addition to age, the other selection criteria include having subjective cognitive concerns; scoring at least 33 in the Telephone Interview for Cognitive Status in Japanese (TICS-J), which indicates no cognitive impairment (Konagaya et al., 2007); available to provide written consent; and available to perform necessary tests on the designated date. The exclusion criteria include neurological impairment, history of previous medications or diseases known to affect the central nervous system, stroke, serious head injury, serious complicating disorder, and under long-term care (defined as those certificated as “care needs levels” or “support need levels” in the public long term care insurance system).

Subjective cognitive concerns (part of the inclusion criteria) were screened by three items of cognitive functioning in the Kihon checklist (KCL) (Sewo Sampaio et al., 2016). The KCL is a self-reporting scale widely used to screen the risk population across Japan, designed by a study group from the Ministry of Health, Labor and Welfare in Japan. Municipalities in Japan have used the KCL to assess and screen high-risk individuals who need intervention, and then provide intervention programs based on its result in each domain to prevent future disability and extend healthy life expectancy. The KCL consists of seven dimensions including daily life, physical strength, nutritional status, oral function, the extent of being housebound, cognitive function, and depression risk. The cognitive function domain in the KCL has three items: (1) “Do your family or your friends point out your memory loss?” (2) “Do you make a call by looking up phone numbers?” and (3) “Do you find yourself not knowing today's date?” (Arai and Satake, 2015). Each item has two choices: “Yes” and “No.” At least one risk response to these three items is considered the risk of cognitive decline and defined as cognitive concerns in this study, referring to the prior RCT studies utilizing KCL to screen the participants with subjective cognitive concern (Huang et al., 2020, 2021).

### 2.2. Intervention

**Figure 2:**
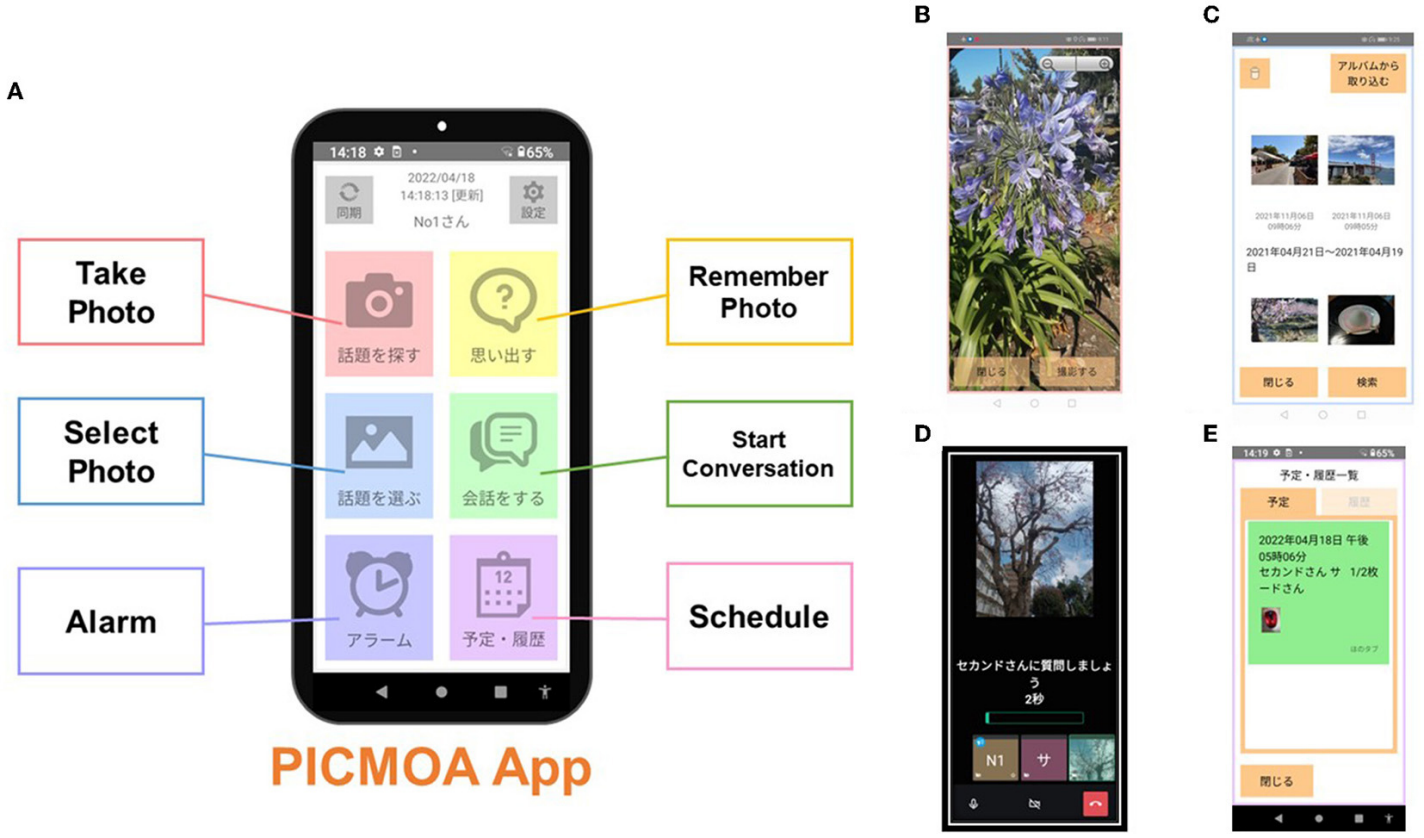
Interface of the PICMOA application. **(A)** Home screen, **(B)** screenshot of “Take Photo” option, **(C)** screenshot of “Select Photo” option, **(D)** screenshot during the conversation session, and **(E)** screenshot of “Schedule” option.

**Figure 3:**
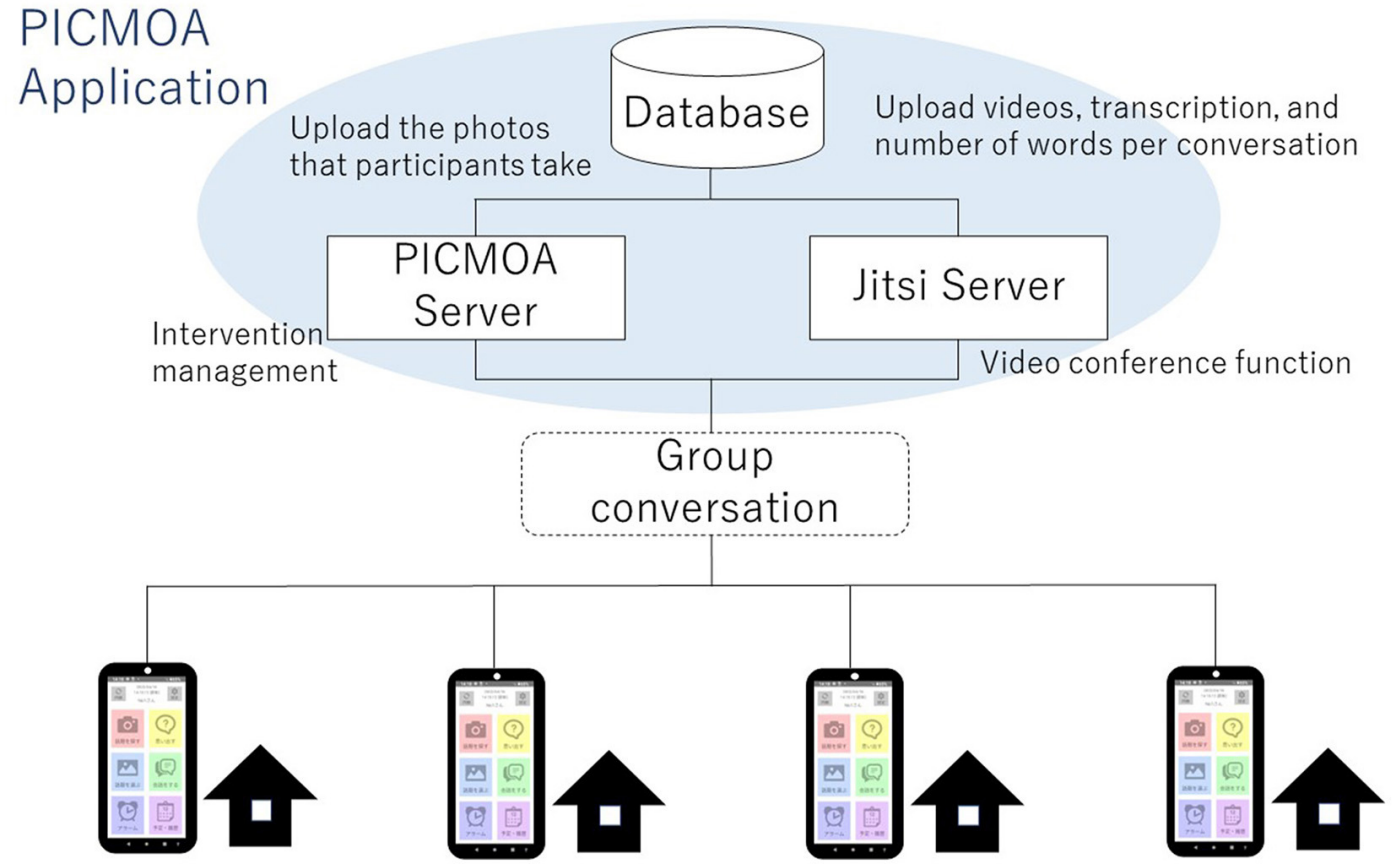
Brief architecture of the PICMOA application.

**Figure 4:**
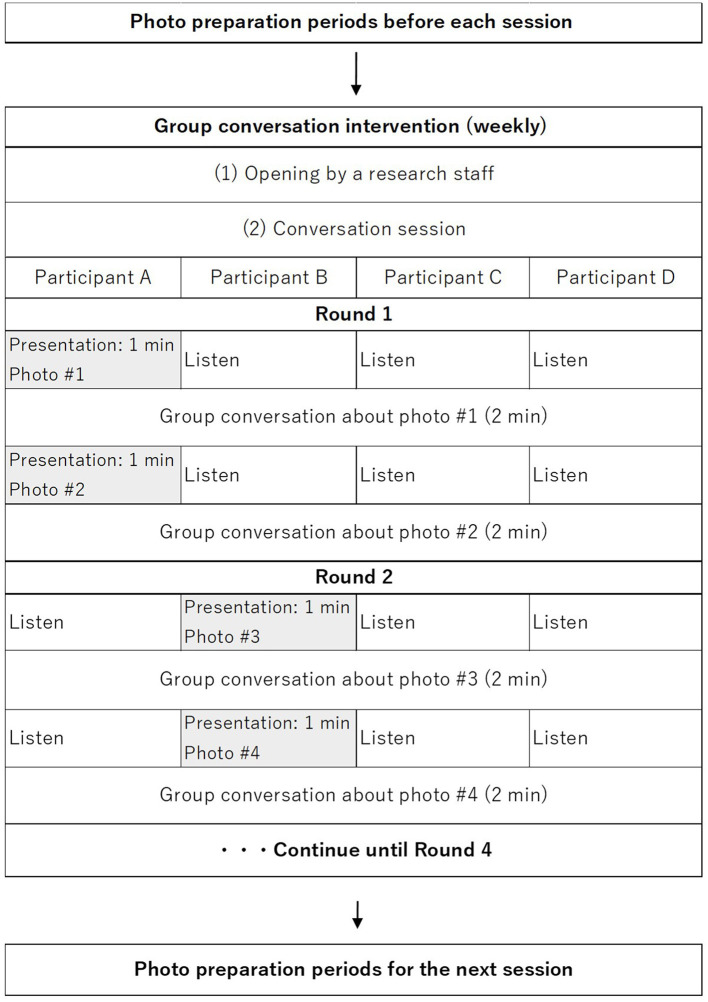
Flow of the structured group conversation during the PICMOA intervention.

The intervention group receives weekly group conversation interventions via smartphone at their home. All participants in the intervention group use PICMOA, which is the smart phone application that provides home-based group conversation for older adults through an easy interface (Figure 2). We prepared the smartphone (Motorola moto g50) with an internet connection and a manual for all participants. The PICMOA application has several functions to facilitate a series of group conversation interventions, including taking photos in daily life, reminiscing about photos taken previously, selecting photos for the intervention session, conversations as intervention sessions, setting an alarm for the intervention, and checking schedules. It was developed based on Jitsi Meet (https://meet.jit.si/) which is an open-source software web conferencing system (Figure 3). The PICMOA intervention was inspired by the PICMOR program, which was a face-to-face group conversation intervention facilitated by a robot used in our previous RCT (Otake-Matsuura et al., 2021). Our series of group conversation interventions consists of (1) a photo preparation period before the session and (2) structured group conversation discussing photos taken by participants according to a specific theme. Figure 4 depicts the flow of the structured group conversation during the PICMOA intervention.

During the one-week preparation period for each intervention session, participants are instructed to take as many photos as possible in their daily life according to the theme of conversation for the next intervention session (e.g., favorite things, neighborhood landmarks) using the camera function in the intervention application. The list of 12 themes of conversation was described in our previous paper (Otake-Matsuura et al., 2021). Then, participants are required to register two photos, which they select from the photos they took during the 1-week preparation period via the application for the intervention session.

The participants join the conversation session in a small group of four people. The conversation system in the application is designed to start automatically on the participants' smartphone at the scheduled time. The group conversation program is structured into presentation and discussion parts. First, one participant presents (presenter) one photo for one minute which is displayed on all participants' smartphones through the application. Subsequently, all other participants ask questions about the photo, and the presenter responds to the questions in a natural conversation context for two minutes. Then, another photo is displayed, and participants continue presenting and discussing the new photo. After one participant presents two photos and all the other participants discuss it, the role of presenter is switched to another participant and the same process is repeated so that participants experience two photos per presenter in one intervention session.

If a participant drops out after allocation to the intervention group, the intensity of the intervention, such as the number of pictures, intervention time, and the number of people to talk to, will change. The bias resulting from inconsistent intervention intensities across groups could directly affect the results and effect sizes. Therefore, in cases where a participant drops out, older volunteers or research staff participate in the intervention session as a substitute to control the intensity of the intervention.

On the other hand, control group participants attend an active control, which is a weekly health education video on the tablet (Lenovo YT-X705L). All participants in the control group are required to watch the health education videos about successful aging for approximately 30 minutes via a webinar (https://zoom.us/) once a week for 12 weeks. Health education videos are provided in a noninteractive way with the participants' cameras and microphones turned off throughout.

### 2.3. Timeline and procedure

#### 2.3.1. Timeline

**Figure 5:**
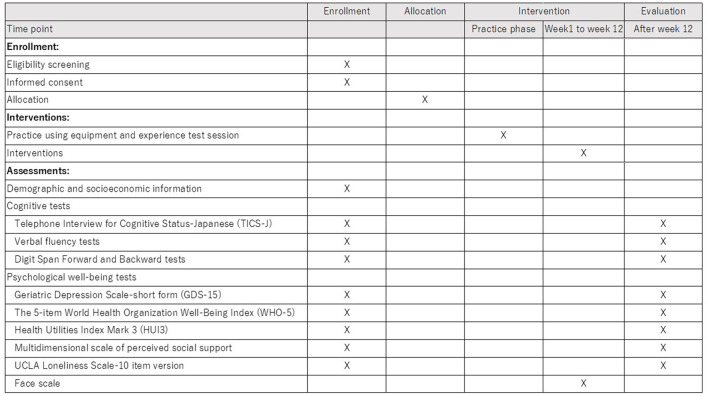
SPIRIT figure – the timeline of the protocol.

The recruitment of participants began in April 2022 and ended in September 2022. The intervention is carried out in two phases (the first half and the second half) considering the feasibility (e.g., equipment). As of November 15, 2022, recruitment was completed, and the second phase of intervention is ongoing. The timeline of the procedure was described as a SPIRIT figure in Figure 5.

#### 2.3.2. Procedure

In the recruitment phase, the municipality government sent recruitment letters to community dwelling older adults, who responded to previous surveys of the municipality and do not have long term care certification, by mailing them in several batches. We also posted flyers on the city's website and placed flyers at city stores. Once our research staffs were contacted by potential participants who expressed their interest in the trial, the eligible participants were required to provide their written informed consent if they agreed to participate after the explanation of the research. The inclusion and exclusion criteria except for the scores of TICS-J were screened at this point.

After obtaining informed consent to participate, questionnaires and standardized neuropsychological batteries were administered as baseline assessments. Based on the scores of TICS-J in the baseline assessment, final screening was done to proceed to the randomization step.

Following the allocation, a letter was sent to each participant to inform them of the group allocation. Next, participants practiced using the equipment (smartphone/ tablet) with the provided manual and timely instruction from research staff, and experienced a test session of PICMOA/health education once. Then, they started the intervention phase for 12 weeks.

#### 2.3.3. Randomization

Stratified randomizations were performed using a permuted block with a 1:1 allocation to one of the two groups: (1) intervention group with PICMOA and (2) control group with health education. Participants were stratified according to sex and sorted based on the TICS-J total scores. Then, permuted block randomization, with a block size of 2, was performed to allocate participants to the group using computer-generated random numbers. The individual in charge of allocation only had access to the following information during the randomization process: ID, TICS-J scores, and sex. All randomization processes were conducted using statistical software R.

### 2.4. Outcomes

#### 2.4.1. Cognitive functioning

The primary endpoint is cognitive functioning at the pre- and post-phase of the 12-week intervention. We hypothesize that PICMOA, i.e., the conversational intervention, may contribute to maintaining or enhancing the linguistic and executive functions among older adults because conversation may be linked to these functions.

To evaluate the intervention effects on cognitive functioning, we have used standardized neuropsychiatry tests. The TICS-J (Konagaya et al., 2007) is used to evaluate global aspects of cognitive functioning. Verbal fluency tests, more specifically, semantic fluency and phonemic fluency tests are introduced to assess retrieval from memory that may also link to executive controlling (Lezak et al., 2012). The verbal fluency test asks to generate the names of animals to test semantic fluency and the words beginning with “ka” to test phonemic fluency. In both tasks, the total number of words produced in one minute is counted. Digit Span Forward and Backward tests from the Wechsler Adult Intelligence Scale – Fourth Edition (WAIS-IV) (Wechsler, 2008) are used for the assessment of simple memory span and working memory capacity, respectively. The Digit Span test requires participants to repeat numbers presented by the examiner in the same order for the Forward test and in reverse order for the Backward test. All neuropsychological test measures are evaluated telephonically by well-trained clinical psychologists. Although this study is an open-label trial, the assessors are blinded for the allocation results to prevent assessment bias.

#### 2.4.2. Psychological well-being

The secondary endpoint evaluates whether the PICMOA intervention effects social and psychological properties including mental status, well-being, loneliness, and social connectedness. The Japanese version of the Geriatric Depression Scale-short form (GDS-15) (Sugishita et al., 2017) is used to assess mental status. The GDS-15 is a common 15-item depression screening scale that showed high validity and reliability among Japanese older population. The score ranges from 0 to 15 with higher scores indicating a more depressed mood. The 5-item World Health Organization Well-Being Index (WHO-5) (Inagaki et al., 2013) is used to assess subjective psychological well-being. The WHO-5 has adequate validity and has been used as an outcome measure in clinical trials (Topp et al., 2015). The percentage score of WHO-5, ranging from 0 to 100, is calculated based on the raw score multiplied by 4. Additionally, the Health Utilities Index Mark 3 (HUI3) (Horsman et al., 2003; Noto and Uemura, 2020) is introduced to evaluate the health-related quality of life (HRQoL). The HUI3 classification system comprises eight components, including vision, hearing, speech, ambulation, dexterity, emotion, cognition, and pain, with five or six levels of ability/disability for each. HUI3 introduces the single- and multi-attribute utility scoring systems. The single-attribute utility score ranges from 0.00 to 1.00 in each component and multi-attribute utility score of overall HRQoL ranges from −0.36 to 1.00.

We use the Multidimensional Scale of Perceived Social Support (MSPSS) (Zimet et al., 1988; Iwasa et al., 2007) and UCLA Loneliness Scale-10 item version (Arimoto and Tadaka, 2019) for social support network and perceived loneliness, respectively, to assess the social aspects. The MSPSS is a 12-item scale. Total score and subscale-scores regarding support from family, friends, and significant others can be calculated by the average of each score, ranging from 0 to 7 where higher score implies stronger support. Loneliness is measured using a 10-item version of the revised UCLA Loneliness Scale which assesses the frequency of feeling lonely with 4 choices per item: (1) never, (2) rarely, (3) sometimes, and (4) always. The total score can range from 10 to 40 with higher score indicating severe loneliness.

During the intervention period, participants are required to answer the 6-point smiley face Likert scale before and after each PICMOA or health education session to evaluate the immediate psychological change.

### 2.5. Sample size

Based on our prior RCT study using group conversation intervention, we hypothesized the small to medium effect size of the PICMOA intervention on the verbal fluency tests, which measure the cognitive outcomes in this study. We used G^*^power (Faul et al., 2007) for sample size calculation, with 80% power, small to medium effect size (f = 0.175), a two-sided hypothesis test, an analysis of variance model between groups over time, and an alpha level of 5%. The sample size calculation showed that we needed a total of approximately 80 participants considering a 15% drop out. Eighty-one participants were included in the trial and allocated to the intervention or control groups.

### 2.6. Data management and monitoring

The research staffs manage all data including the results of neuropsychiatry tests and questionnaires. Data entry will be outsourced to a third party. All records of nonadherence and drop-out will be electronically documented. Anonymized data stored on a secure computer will be used for analysis, and the data with personal information will be kept in a locked cabinet in a secured office.

### 2.7. Statistical analyses

To estimate the effect of the PICMOA intervention, the linear mixed models with random intercepts for outcome measurements will be computed. The models will include the following independent variables: groups assignment factor (PICMOA group: 1, control group: 0), time factor (pre: 0, and post: 1), and their interaction term (time × group). The interaction term will be interpreted as the intervention effect. Given that some baseline statuses such as cognitive functioning, psychosocial status, and familiarity with using equipment may influence the effect of this social intervention, we will examine how much the intervention effects vary depending on the levels of these statuses.

All analyses will be performed using R software, and statistical significance threshold will be set as *P* < 0.05.

### 2.8. Ethics

This study was approved by the ethics committee of RIKEN (RIKEN-W3-2021-049). Written informed consent was obtained from all the participants. All procedures are conducted in accordance with the ethical principles for medical research involving human subjects detailed in the Declaration of Helsinki.

## 3. Discussion

While there is an increasing focus on internet-based social activity intervention with the development of communication technology in recent years, the impact of remote social activity intervention on cognitive functioning is still uncertain. We developed PICMOA, which is a home-based group conversation intervention, with a focus on the close connection between linguistic ability and cognitive health. Although our PICMOA trial is underway for evaluation, it is expected to accumulate evidence with the following strengths.

First, PICMOA allows participants to join sessions from home or anywhere. While old age is a period when accessibility tends to decline due to various functional declines and environmental changes, this application provides participants with greater accessibility to preventative interventions. Second, the structured group conversation program which is the content of the PICMOA intervention gives participants equal opportunities to talk about their own photos and ask questions. The amount of speech during the session may lead to less biases among participants, which means that the intensity of intervention would be more uniform compared to general conversation. Third, the PICMOA application has technological strength as it has been developed to be both user- and interventionist-friendly. This application has a user-friendly interface that is easy to use, and the intervention session automatically starts at the predetermined intervention time by utilizing the scheduler and signaling the server in the application system. This enables participants, even older adults who do not touch their smartphones regularly, to join the intervention session easily, without having to launch the application to access the intervention session. Many older people are not familiar with operations on digital devices such as smartphones yet, but this application is designed to be user-friendly. Furthermore, PICMOA has an automatic moderator function that, in an artificial voice, facilitates conversations by moderating the presenters' turns and switching photos based on the predetermined time slot duration. This system may contribute to conserving human resources, especially in the facilitation of interventions and time management that is carried out by humans in classic social interventions. Fourth, this trial includes older adults at risk of cognitive decline which is operationally defined as subjective cognitive concern using the KCL. Individuals at risk of cognitive decline screened by KCL are the target of preventive care in the public service (long-term care and living support project) in Japan; targeting this population in the trial has great public health importance.

The limitations of the study should also be noted. Although a large-scale trial is ideal for sub-analysis such as stratified analysis by various demographics, this trial has been conducted with the required sample size of over 80 people due to feasibility. Additionally, this ongoing trial cannot address the neural mechanism of the intervention effect because we did not plan to collect brain-related information, such as MRI data to prioritize having less direct contact with participants for their safety.

Despite these limitations, we believe that our ongoing trial can contribute by adding new knowledge regarding the impact of remote social activity intervention. As the global burden of dementia and the serious situation of social isolation are important public health issues, our intervention, which utilizes remote technology, will help by providing an avenue for preventive care to an isolated population and introducing a new way of social bonding and community participation. We are accumulating evidence regarding the effects of the intervention in the ongoing RCT and plan to confirm its applicability and issues in future large-scale trials.

## Ethics statement

The studies involving human participants were reviewed and approved by the Ethics Committee of RIKEN. The patients/participants provided their written informed consent to participate in this study.

## Author contributions

KM contributed to the conception, study design, drafting, and revision of this paper. ST, TS, and HS assisted in the conception, drafting, and revision of this paper. MO-M is the principal investigator and designed the smartphone application. All authors have read and approved the final manuscript.

## References

[B1] Amieva H. Stoykova R. Matharan F. Helmer C. Antonucci T. C. Dartigues J. F. (2010). What aspects of social network are protective for dementia? Not the quantity but the quality of social interactions is protective up to 15 years later. Psychosom. Med. 72, 905–911. 10.1097/PSY.0b013e3181f5e121 20807876

[B2] Arai H. Satake S. (2015). English translation of the Kihon Checklist. Geriatr. Gerontol. Int. 15, 518–519. 10.1111/ggi.12397 25828791

[B3] Arimoto A. Tadaka E. (2019). Reliability and validity of Japanese versions of the UCLA loneliness scale version 3 for use among mothers with infants and toddlers: a cross-sectional study. BMC. Womens. Health. 19,105. 10.1186/s12905-019-0792-4 31349835 PMC6660924

[B4] Asgari M. Kaye J. Dodge H. (2017). Predicting mild cognitive impairment from spontaneous spoken utterances. Alzheimers. Dement. 3, 219–228. 10.1016/j.trci.2017.01.006 29067328 PMC5651423

[B5] Dodge H. H. Zhu J. Mattek N. C. Bowman M. Ybarra O. Wild K. . (2015). Web-enabled conversational interactions as a method to improve cognitive functions: results of a 6-week randomized controlled trial. Alzheimers. Dement. 1, 1–2. 10.1016/j.trci.2015.01.001 26203461 PMC4507295

[B6] Faul F. Erdfelder E. Lang A.-G. Buchner A. (2007). G^*^Power 3: a flexible statistical power analysis program for the social, behavioral, and biomedical sciences. Behav. Res. Methods. 39, 175–191. 10.3758/BF03193146 17695343

[B7] Forbes-McKay K. Shanks M. F. Venneri A. (2013). Profiling spontaneous speech decline in Alzheimer's disease: a longitudinal study. Acta. Neuropsychiatr. 25, 320–327. 10.1017/neu.2013.16 25287871

[B8] Fraser K. C. Meltzer J. A. Rudzicz F. (2016). Linguistic features identify Alzheimers disease in narrative speech. J. Alzheimers. Dis. 49, 407–422. 10.3233/JAD-150520 26484921

[B9] Fratiglioni L. Wang H. X. Ericsson K. Maytan M. Winblad B. (2000). Influence of social network on occurrence of dementia: a community-based longitudinal study. Lancet. 355, 1315–1319. 10.1016/S0140-6736(00)02113-9 10776744

[B10] Horsman J. Furlong W. Feeny D. Torrance G. (2003). The health utilities index (HUI): concepts, measurement properties and applications. Health. Qual. Life. Outcomes. 16,54. 10.1186/1477-7525-1-54 14613568 PMC293474

[B11] Huang C. H. Umegaki H. Makino T. Uemura K. Hayashi T. Kitada T. . (2020). Effect of various exercises on frailty among older adults with subjective cognitive concerns: a randomised controlled trial. Age. Ageing. 49, 1011–1019. 10.1093/ageing/afaa086 32520984

[B12] Huang C. H. Umegaki H. Makino T. Uemura K. Hayashi T. Kitada T. . (2021). Effect of various exercises on intrinsic capacity in older adults with subjective cognitive concerns. J. Am. Med. Dir. Assoc. 22, 780–786. 10.1016/j.jamda.2020.06.048 32768376

[B13] Inagaki H. Ito K. Sakuma N. Sugiyama M. Okamura T. Awata S. (2013). Reliability and validity of the simplified Japanese version of the WHO-Five wellbeing index (S-WHO-5-J). Nihon. Koshu. Eisei. Zasshi. 60, 294–301. In Japanese. 10.11236/jph.60.5_294 23942026

[B14] Iwasa H. Gondo Y. Masui Y. Inagaki H. Kawaai C. Otsuka R. . (2007). Reliability and validity of Japanese version of multidimensional scale of perceived social support. J. Health Welfare Stat. 54, 26–33. In Japanese.

[B15] Karp A. Paillard-Borg S. Wang H. X. Silverstein M. Winblad B. Fratiglioni L. (2006). Mental, physical and social components in leisure activities equally contribute to decrease dementia risk. Dement. Geriatr. Cogn. Disord. 21, 65–73. 10.1159/000089919 16319455

[B16] Konagaya Y. Washimi Y. Hattori H. Takeda A. Watanabe T. Ohta T. (2007). Validation of the telephone interview for cognitive status (TICS) in Japanese. Int. J. Geriatr. Psychiatry. 22, 695–700. 10.1002/gps.1812 17431929

[B17] Kuiper J. S. Zuidersma M. Oude Voshaar R. C. Zuidema S. U. van den Heuvel E. R. Stolk R. P. . (2015). Social relationships and risk of dementia: a systematic review and meta-analysis of longitudinal cohort studies. *Ageing Res*. Rev. 22, 39–57. 10.1016/j.arr.2015.04.006 25956016

[B18] Lezak M. D. Howieson D. B. Bigler E. D. Tranel D. (2012). Neuropsychological Assessment. Oxford: Oxford University Press.

[B19] Livingston G. Huntley J. Sommerlad A. Ames D. Ballard C. Banerjee S. . (2020). Dementia prevention, intervention, and care: 2020 report of the Lancet Commission. Lancet. 396, 413–446. 10.1016/S0140-6736(20)30367-6 32738937 PMC7392084

[B20] Mueller K. D. Koscik R. L. Hermann B. P. Johnson S. C. Turkstra L. S. (2018). Declines in connected language are associated with very early mild cognitive impairment: results from the wisconsin registry for Alzheimer's prevention. Front. Aging Neurosci. 9, 437. 10.3389/fnagi.2017.00437 29375365 PMC5767238

[B21] Murayama H. Okubo R. Tabuchi T. (2021). Increase in Social Isolation during the COVID-19 pandemic and its association with mental health: findings from the JACSIS 2020 Study. Int. J. Environ. Res. Public. Health. 18, 8238. 10.3390/ijerph18168238 34443988 PMC8394951

[B22] Noto S. Uemura T. (2020). Japanese health utilities index mark 3 (HUI3): measurement properties in a community sample. J. Patient. Rep. Outcomes. 4, 9. 10.1186/s41687-020-0175-5 31997027 PMC6987883

[B23] Otake-Matsuura M. Tokunaga S. Watanabe K. Abe M. S. Sekiguchi T. Sugimoto H. . (2021). Cognitive intervention through photo-integrated conversation moderated by robots (PICMOR) program: a randomized controlled trial. Front. Robot. AI. 8, 633076. 10.3389/frobt.2021.633076 33969003 PMC8103544

[B24] Saczynski J. S. Pfeifer L. A. Masaki K. Korf E. S. Laurin D. White L. . (2006). The effect of social engagement on incident dementia: the Honolulu-Asia aging study. Am. J. Epidemiol. 163, 433–440. 10.1093/aje/kwj061 16410348

[B25] Sepúlveda-Loyola W. Rodríguez-Sánchez I. Pérez-Rodríguez P. Ganz F. Torralba R. Oliveira D. V. . (2020). Impact of social isolation due to COVID-19 on health in older people: mental and physical effects and recommendations. *J. Nutr. Health*. Aging. 24, 938–947. 10.1007/s12603-020-1500-7 PMC7597423 33155618

[B26] Sewo Sampaio P. Y. Sampaio R. A. Yamada M. Arai H. (2016). Systematic review of the Kihon Checklist: Is it a reliable assessment of frailty? Geriatr. Gerontol. Int. 16, 893–902. 10.1111/ggi.12833 27444395

[B27] Sugimoto H. Kawagoe T. Otake-Matsuura M. (2020). Characteristics of resting-state functional connectivity in older adults after the PICMOR intervention program: a preliminary report. BMC. Geriatr. 20,486. 10.1186/s12877-020-01892-2 33218309 PMC7678164

[B28] Sugimoto H. Otake-Matsuura M. (2022a). A pilot voxel-based morphometry study of older adults after the PICMOR intervention program. BMC. Geriatr. 22, 63. 10.1186/s12877-021-02669-x 35045810 PMC8772081

[B29] Sugimoto H. Otake-Matsuura M. (2022b). Tract-based spatial statistics analysis of diffusion tensor imaging in older adults after the PICMOR intervention program: a pilot study. Front. Aging. Neurosci. 14, 867417. 10.3389/fnagi.2022.867417 35721023 PMC9204185

[B30] Sugishita K. Sugishita M. Hemmi I. Asada T. Tanigawa T. (2017). A validity and reliability study of the Japanese version of the geriatric depression scale 15 (GDS-15-J). Clin. Gerontol. 40, 233–240. 10.1080/07317115.2016.1199452 28452641

[B31] Taler V. Phillips N. A. (2008). Language performance in Alzheimer's disease and mild cognitive impairment: a comparative review. J. Clin. Exp. Neuropsychol. 30, 501–556. 10.1080/13803390701550128 18569251

[B32] Topp C. W. Østergaard S. D. Søndergaard S. Bech P. (2015). The WHO-5 wellbeing index: a systematic review of the literature. Psychother. Psychosom. 84, 167–176. 10.1159/000376585 25831962

[B33] Wako city (2021). Population statistics. Available online at: http://www.city.wako.lg.jp/home/shisei/toukei/you_2_13/_20610/_20626.html (accessed November 9, 2022).

[B34] Wechsler D. (2008). Wechsler Adult Intelligence Scale—Fourth Edition. San Antonio: Pearson Assessment. 10.1037/t15169-000

[B35] World Health Organization (2019). Risk reduction of cognitive decline and dementia—WHO Guidelines. Available online at: https://www.who.int/mental_health/neurology/dementia/guidelines_risk_reduction/en/ (accessed June 7, 2022). 31219687

[B36] Ybarra O. Burnstein E. Winkielman P. Keller M. C. Manis M. Chan E. . (2008). Mental exercising through simple socializing: social interaction promotes general cognitive functioning. Pers. Soc. Psychol. Bull. 34, 248–259. 10.1177/0146167207310454 18212333

[B37] Ybarra O. Winkielman P. (2012). On-line social interactions and executive functions. Front. Hum. Neurosci. 6, 75. 10.3389/fnhum.2012.00075 22509160 PMC3321651

[B38] Zimet G. D. Dahlem N. W. Zimet S. G. Farley G. K. (1988). The multidimensional scale of perceived social support. J. Pers. Assess. 52, 30–41. 10.1207/s15327752jpa5201_2 2280326

